# Engaging primary care providers in managing pediatric eating disorders: a mixed methods study

**DOI:** 10.1186/s40337-020-00363-8

**Published:** 2021-01-14

**Authors:** Jocelyn Lebow, Cassandra Narr, Angela Mattke, Janna R. Gewirtz O’Brien, Marcie Billings, Julie Hathaway, Kristin Vickers, Robert Jacobson, Leslie Sim

**Affiliations:** 1 Department of Psychiatry and Psychology, Mayo Clinic, Mayo Clinic School of Medicine, 200 First Street SW, Rochester, MN 55905 USA; 2 Mayo Clinic Robert D. and Patricia E. Kern Center for the Science of Health Care Delivery, Rochester, MN USA; 3 Department of Pediatric and Adolescent Medicine, Mayo Clinic School of Medicine, Rochester, MN USA; 4 Department of Pediatrics, University of Minnesota, Minneapolis, MN USA

**Keywords:** Anorexia nervosa, Bulimia nervosa, Eating disorder, Primary care, Adolescent

## Abstract

**Background:**

The primary care setting offers an attractive opportunity for, not only the identification of pediatric eating disorders, but also the delivery of evidence-based treatment. However, constraints of this setting pose barriers for implementing treatment. For interventions to be successful, they need to take into consideration the perspectives of stakeholders. As such, the purpose of this study was to examine in-depth primary care providers’ perspective of challenges to identifying and managing eating disorders in the primary care setting.

**Methods:**

This mixed methods study surveyed 60 Pediatric and Family Medicine providers across 6 primary care practices. Sixteen of these providers were further interviewed using a qualitative, semi-structured interview.

**Results:**

Providers (*n* = 60, response rate of 45%) acknowledged the potential of primary care as a point of contact for early identification and treatment of pediatric eating disorders. They also expressed that this was an area of need in their practices. They identified numerous barriers to successful implementation of evidence-based treatment in this setting including scarcity of time, knowledge, and resources.

**Conclusions:**

Investigations seeking to build capacities in primary care settings to address eating disorders must address these barriers.

**Supplementary Information:**

The online version contains supplementary material available at 10.1186/s40337-020-00363-8.

## Plain English summary

The primary care setting offers an attractive opportunity for, not only the identification of eating disorders in children and adolescents, but also the delivery of interventions that can shorten the course of these life-threatening illnesses. However, due to the unique nature of primary care, effective interventions must be designed specifically to fit within this setting. As such, the purpose of this study was to examine primary care providers’ perspective of challenges to identifying and managing eating disorders in the primary care setting. We surveyed 60 Pediatric and Family Medicine providers across 6 primary care practices. Sixteen of these providers were further questioned using more in-depth interview. Providers acknowledged the potential of primary care as a point of contact for early identification and treatment of pediatric eating disorders. They also expressed that this was an area of need in their practices. They identified numerous barriers to successful treatment in this setting including limited time, knowledge, and resources. Researchers seeking to build eating disorder interventions in primary care settings must address these numerous barriers.

## Introduction

Two of the highest priorities in the field of pediatric eating disorders are early identification and expanding access to evidence-based treatment [1]. Because delayed detection and treatment are associated with poor prognosis, prompt access to care is vital [2–4]. The substantial physical morbidity and mortality associated with eating disorders makes this agenda especially urgent [5]. Given the impact of the global COVID-19 pandemic on increasing rates and severity of eating disorders, the need for expanded options to identify eating disorders and access care is particularly salient [6].

Primary care offers several advantages for improving efficient and effective treatment of child and adolescent eating disorders. The majority of young patients with eating disorders present first to their primary care provider, often with complaints such as abdominal pain, fatigue, or menstrual abnormalities [7]. Although previous research suggests that primary care providers converge in identifying eating disorders as an area of concern, they express varying degrees of comfort with identification and management of these patients [8], and diverge on whether they feel this identification and management is within their scope of practice [9]. This discrepancy may lead to missed opportunities for early identification and intervention.

When pediatric and family medicine providers are given the expertise and direction to include eating disorders on their differential, they are well-positioned to identify these potentially life-threatening disorders early in the illness trajectory. In addition, primary care providers are well-suited to address patient and parent ambivalence in accepting an eating disorder diagnosis, a common barrier to treatment. Given the expertise of primary care providers in coaching families to engage in difficult but essential health behaviors (e.g. vaccinations, inhaler use, medication adherence), they are well-equipped to help patients and families transition from pre-contemplation to action in implementing behavior change.

Primary care providers may also bolster adherence through their often-longstanding relationships with families that can be leveraged to efficiently move families towards accepting the eating disorder diagnosis and preparing them to engage in intervention. In particular, research suggests that primary care providers’ conversations with patients about health behaviors are linked to patient confidence in their ability to follow through on treatment recommendations [10]. As such, primary care providers may have a distinct ability to mobilize families to begin the lengthy and difficult process of addressing their child’s eating disorder.

In spite of such advantages, implementation of eating disorder screening and intervention in primary care is complicated by numerous barriers, including scarcity of resources and time, as well as physician confidence and/or interest in treating these disorders [11]. Previous surveys of primary care physicians across the United States and Canada have found while up to 91% of pediatricians and family physicians feel that it is their role to assess for and identify eating disorders, they often fail to identify these conditions [9, 12]. For example, one study found that 68% of primary care providers only screened for eating disorders when it was associated with the patient’s specific presenting problem [11].

When asked about eating disorder intervention, primary care providers were even less confident, with less than half reporting they felt they had the necessary skills to intervene with these disorders in their practice [11, 13]. In one study surveying a group of 70 pediatricians, only 21.5% reported feeling comfortable providing treatment for eating disorders [13]. In fact, the number of pediatricians who felt comfortable treating eating disorders was lower than for any other behavioral health problem including anxiety, depression, somatic symptom disorders, and attention deficit/hyperactivity disorder [13]. With regards to primary care training, less than half of family medicine and pediatric residents report receiving what they feel is adequate training in eating disorders during residency, and over 70% in each group expressed a desire to have more training in this area [14].

In order to successfully leverage the resources of the primary care setting, we must engage primary care provider stakeholders to help identify and address critical system-wide barriers. Though there have been several surveys of primary care providers’ needs, comfort, and preferences in diagnosing and/or treating eating disorders, all these studies have been restricted to broad quantitative surveys, which limits a deeper understanding of opportunities for system-wide changes to enhance the identification and treatment of this population within primary care.

This mixed method study is the first attempt to clarify the perspectives of primary care providers on the identification and treatment of eating disorders in their practices using the combination of a broad survey with in-depth follow-up interviews to assess the attitudes and opinions of primary care providers representing both Family Medicine and Pediatrics, including physicians, nurse-practitioners and physician’s assistants. Questions focused on their assessment of the need and feasibility for eating disorder care in a primary care setting, their level of confidence for managing children and adolescents with eating disorders in their practice, their view on what resources were needed to assist these patients in primary care, and their opinions on barriers that currently exist to providing effective management.

## Methods

All primary care providers in six Midwestern practices who treated children (*N* = 132) were contacted to participate in a survey regarding managing pediatric eating disorders in their daily work. The primary care practices were all associated with an academic medical center and consisted of 5 separate clinics located in a midsize city and one clinic located in a rural community approximately 10 miles outside of the city. Along with a consent document, the paper survey was sent to 132 providers in the Division of Community Pediatrics and Adolescent Medicine (CPAM) and the Department of Family Medicine. Of these providers, 60 (45%) completed the survey. Primary care providers responded to a survey item regarding their willingness to participate in an in-person semi-structured interview to assess perceived needs and barriers for treating eating disorders within primary care. Twenty-five (42%) stated they would be willing to participate and, of these individuals, 16 providers participated in the semi-structured interviews before theoretical saturation [15] was reached.

The number of patients with an eating disorder that each provider estimated they saw each year varied widely from 2 to 15. Given the variability in experience and skills to identify eating disorders, this is not likely to represent the true prevalence of eating disorders but instead reflects how many they suspect present to their practice. The study was approved by the Mayo Clinic Institutional Review Board.

### Measures

Recognizing the numerous competing demands on primary care providers’ time, the 8-item quantitative survey was designed to be brief. Questions included Likert-scale ratings of confidence in identifying, medically managing, and advising families around decision-making for the treatment of pediatric eating disorders, as well as a question about the makeup of their patient panel. Primary care providers were also asked to evaluate a case study of a young patient with an eating disorder and discuss their clinical response in an additional item. Finally, providers were asked about their interest in new tools and strategies for addressing disordered eating in their practices. Since no reliable and valid tools exist to assess knowledge or confidence in treating eating disorders among primary care providers, based on a review of the literature, experts in the fields of eating disorders and qualitative research (J.L, L.S., K.V.) developed survey items and interview questions [8, 9, 11–14].

Provider interviews were conducted in-person by an experienced qualitative interviewer (JH) who was not involved with the primary care providers’ clinical practice, and took an average of 15–20 min. The interviewer followed a semi-structured interview script that was designed with consideration of guidelines for minimizing bias and increasing the reliability and validity of interview data [16, 17]. Questions for primary care providers included close-ended prompts designed to gauge the provider’s familiarity and comfort-level with eating disorder management and assessment, as well as open-ended questions regarding their needs, preferences, and potential barriers to be considered in the development of a primary-care based eating disorder intervention. Interviews were audio-recorded and transcribed verbatim. This interviewer also kept concurrent field notes to help with transcript interpretation. See Supplementary Material for both the quantitative survey and qualitative interview guide.

### Analysis

Quantitative analysis of survey items was completed, including descriptive statistics appropriate for this level of data, using IBM Statistical Package for the Social Sciences, version 25. Qualitative data were coded using thematic analysis to identify core categories. Dominant themes, as determined based on multiple participants repeating similar experiences, attitudes, or needs, were identified and a coding strategy was developed. Two investigators (KV, JL) coded all qualitative interviews using content analysis, a systematic process of deductively sorting information based on previously identified themes [18, 19]. After investigators independently coded all qualitative data, predominant categories were agreed upon and representative quotes were identified. When discrepancies in coding occurred, the two analysts met and reviewed the full interview transcript to better understand meaning through examination of the original context.

## Results

### Quantitative data: brief provider perspective survey

Sixty out of the 132 primary care providers contacted (45% response rate) completed the 8-item survey examining self-reported confidence and interest in delivering eating disorder treatment in the primary care setting (Table 1). Participants included 14 providers from CPAM (23.3%), 37 from Family Medicine (61.7%) and 9 providers who declined to report their Department. In terms of education and role, 36 were physicians (MD or DO; 60%), 20 were nurse practitioners (33.3%), and 4 were physician assistants (6.7%,).

**Table 1:** Primary Care Provider Confidence and Interest in Delivering Eating Disorder Treatment (*N* = 60)

At this time …	Mean (SD)
How confident are you in your ability to evaluate a patient for the presence of disordered eating in your daily practice? (0 = Not very confident; 10 = Very confident)	5.53 (2.09)
How confident are you in your ability to manage disordered eating in your daily practice? (0 = Not very confident; 10 = Very confident)	3.68 (2.20)
How confident are you in your ability to provide diagnostic feedback to families about the presence of an eating disorder/disordered eating in your daily practice? (0 = Not very confident; 10 = Very confident)	4.34 (2.26)
How confident are you in your ability to advise and support a patient and their family in making decisions about his/her eating disorder treatment? (0 = Not very confident; 10 = Very confident)	4.15 (2.11)
How interested would you be in learning new tools or strategies for addressing disordered eating in your practice? (0 = Not very interested; 10 = Very interested)	8.52 (1.83)
How interested would you be in the development of a new ICS [Integrated Community Specialty: specialists embedded in the practice] for addressing eating and weight disorders? (0 = Not very interested; 10 = Very interested)	8.81 (1.82)

** 2 additional items included a case example and a question about patient panel makeup

On a 10-point scale, where scores less than 5 were considered low, scores between 5 and 8 were considered moderate, and scores above 8 considered high, primary care providers endorsed moderate confidence in their ability to evaluate a patient for an eating disorder (mean 5.53, SD 2.09), but low confidence in their ability to manage disordered eating in pediatric patients in their practices (means < 5 for all responses, SDs 2.11–2.26). Respondents rated themselves highly (means > 8, SDs 1.82–1.83) with respect to interest in learning new tools or strategies for eating disorder treatment in their clinical area and also in working with an eating disorder specialist integrated into their practice.

### Qualitative data predominant themes: resource needs and feasibility concerns

Sixteen primary care providers (13 females, 81%) participated in interviews including 10 from Family Medicine and 6 from CPAM. Of these providers, 11 were physicians, and 5 were either nurse practitioners or physician assistants.

Interviews from these 16 providers revealed themes consistent with the survey data, in that providers value eating disorder treatment, have low confidence in their own ability to manage eating disorders in their clinical setting, and would be interested in receiving support from eating disorder experts in managing their patients with these complex problems. However, providers identified significant challenges providing care for patients with disordered eating within the constraints of the existing system. Here we organize the predominant themes in two sections. The first set of themes demonstrate provider recognition that current identification and care of pediatric patients with eating disorders is suboptimal in their primary care setting related to current practice barriers. The second set of themes reveal the complexities of successfully implementing and maintaining a new model of care for treatment of pediatric eating disorders in primary care. The resounding primary themes across all interviews and woven within all identified sub-themes are the debilitating impact of insufficient time and the competing demands for that limited time, as experienced by all primary care providers interviewed.

#### Current barriers to optimal eating disorder management in primary care

Providers described personal, professional, and system-level issues that contribute to a status quo of sub-optimal identification and treatment of pediatric eating disorders, as well as the complexities associated with these disorders themselves. Most providers do not feel there is sufficient time or a simple process to identify young patients with eating disorders: “In primary care, we have X amount of time with patients and our nurses have multiple things to screen for. There’s educational factors, there’s exercise, there’s tobacco exposures, there’s health exposures, there’s screen time. There are so many things to screen for that we end up diluting what we do effectively. So, time is the enemy in multiple screening. How do you decide to spend your time screening for eating disorders versus convincing them to get HPV vaccination?” (participant 014). Some feel they lack sufficient knowledge to intensify their treatment of these patients, and given limited time and the perception of eating disorders being a less frequent problem, they focus on other important mental health and physical health issues. None diminish the importance of eating disorder treatment: “Honestly probably not interested in training on this. Just that I’m stretched too thin right now and I’m working on saying no to things. I care about this issue and I would like to see better services for our patients.” (participant 001).

In contrast to an existing process at 3 of these 6 practices for screening adolescents with depression and connecting them to onsite treatment, there is no systematic approach to eating disorder identification or treatment in their setting. Primary care providers view eating disorder management as complex, and cite aspects such as challenging family dynamics, patients resisting treatment, and provider concerns about the potential to harm the patient/provider relationship if a diagnosis or treatment is pushed: “It’s very difficult to have success with people with eating disorders” (participant 013); “It’s something that we are not specifically trained in;” and “It’s super emotional and can get ugly and there’s that impact on the trust and relationship” (participant 001).

Further, if patients do require specialty care, there is a troubling lack of local treatment options. In fact, primary care providers explained that local eating disorder treatment options have decreased, with the few specialists unable to meet the current demand. There is an implicit theme of emotional exhaustion among providers at the thought of trying to do more for these complex patients as a non-specialist when access to more intensive, medically-necessary treatment is unavailable.

#### Future challenges in implementing system change for pediatric eating disorder management in primary care setting

Though providers appreciated the importance of eating disorder identification and treatment, they expressed mixed views about the feasibility of a program meant to support eating disorder treatment in primary care. Some primary care providers expressed an interest in an integrated model of care, in which providers would receive training in eating disorders and then provide direct care in collaboration with a specialist: “Anything. I’d be interested in anything. Lectures, conferences, webinars.” (participant 015).

Though many primary care providers recognized a need to better serve patients with eating disorders, most providers described ambivalence, and some were pessimistic about model implementation without addressing key barriers, such as inadequate time and limited resources.” I guess we could do it but we can’t do it without scheduled time. We already have so much that we do that is not scheduled that it’s ridiculous already.” (participant 012). Some expressed concerns about compatibility with the workflow and the relative advantage of implementing a new program, rather than continuing with the status quo of referring elsewhere, “It’s not completely reasonable honestly and it concerns me. If the choice is nothing or that, then it’s probably a good thing to do... or let the [established eating disorder program 90 miles away provide eating disorder care].” (participant 001). Others suggest that there are a few providers that want to do more of this work: “I think probably like three of us that are working on it or interested. There might be a couple more.” (participant 015).

Although not a major theme, some providers made comparisons to existing programs in primary care that include the integrated care program for adolescent depression, and there was appreciation for standardized, brief screening tools and onsite support. Others say that these same resources are spread too thin currently, and access remains a problem, which may contribute to lack of enthusiasm for a new care model for eating disorder treatment. Sustainability of a new program and affordability to patients were also mentioned as concerns.

## Discussion

This mixed methods study is the first to take an in-depth look at the attitudes, perceived needs, and barriers primary care providers face in implementing screening and/or eating disorder management in primary care. These results suggest that, in line with previous broad-based survey studies [11, 12], though providers are aware that they need additional support in addressing eating disorders, they are frustrated by the lack of resources and real barriers to care, such as competing demands for limited time and lack of processes to best serve young patients with eating disorders. They emphasize that implementation of any primary care-based eating disorder interventions must be thoughtful and strategic, or risk repeating failures of past programs.

Similar to previous studies, primary care providers reported that they lack the time, training, and skills to provide or access appropriate eating disorder care for their patients. Providers are willing to follow guidelines, but have limited awareness regarding clinical best practices. Also resonant with other surveys [9, 11, 13], they endorse low levels of confidence in their ability to identify and manage eating disorders, which they view as complex and challenging illnesses. Although they endorse a strong desire to improve their skills to help these patients, they identify numerous barriers to doing so, including limited time for provider training and implementation. Finally, providers expressed concern about the lack of validated, effective, and efficient screening tools for pediatric patients, as well as concerns about the realities of implementing any type of universal screening in their already overburdened and brief visits. Though eating disorder screeners exist, most (e.g. the SCOFF) [20] are not validated for pediatric patients. The few screeners that are validated for younger populations (e.g. the Eating Attitudes Test (EAT-26) [21]) are relatively lengthy for integration into a busy primary care practice, especially at the level of universal screening.

The lack of eating disorder resources was a source of significant dissatisfaction for providers. They expressed negative emotion around the lack of resources including shame and frustration that, even at a well-known, highly-resourced institution, there were few treatment options to offer pediatric patients with eating disorders. They also discussed the tremendous burden this places on patients and their families of having to seek treatment at an outside program, frequently outside the region or state.

There was consensus that the current status of eating disorder resources is insufficient to care for young patients in their community. Primary care providers expressed a clear desire to help these patients and a need for support to do so. Because they frequently encountered these patients in their practice, they believed that the primary care setting has considerable potential for the early intervention of these conditions. Yet, they stated for this to happen, the intervention must fit within the constraints of the practice. In the context of the current system, a primary care-based program for pediatric eating disorders might be at risk for failure were it not to consider these barriers during planning and implementation. In other words, primary care providers voiced a rational ambivalence about offering screening, intervention, and management of eating disorders in primary care. Although they feel this is an important and an underserved area, they are concerned about stretching their already overtaxed resources to address these complex and challenging patients, without practice-level changes and supports.

This is particularly important at present. On one hand, the global COVID-19 pandemic has led to increased numbers of patients with eating disorders struggling to access care [6], and thus, there is great need for additional avenues to identify and treat patients with eating disorders, such as could be implemented through primary care. On the other hand, however, the pandemic has exponentially multiplied the demands on health care providers’ time, energy, and other resources. For programs to be successful, it is important for the specialist hoping to enter the primary care setting to understand the landscape. Specialists need to address the actual barriers to eating disorder management, including the scarcity of time, provider discomfort with the illness, and the perceived complexity of the disorder. It is tempting for eating disorder specialists to assume the main barrier to primary care-based eating disorder care is a knowledge gap, and that effective intervention will begin (and potentially end) with psychoeducation about eating disorder detection and management. This lack of cultural humility gravely misunderstands the actual barriers in primary care, and, at best, will result in the provision of redundant information to a group that is already aware of the dangers of eating disorders. At worst, knowledge-based interventions run the risk of increasing already-present feelings of guilt and shame in primary care providers without providing any sort of workable solution.

Primary care is not a setting where it is feasible for a specialist to enter, implement an intervention program (even one with a strong evidence base), and leave. Workable solutions begin with relationship-building among stakeholders, and should be informed by implementation science. It is not sufficient to attempt to implement a model that has worked in a specialty setting; specialists must spend time developing an innovative intervention that is effective and sustainable within the primary care system.

In general, possessing onsite resources greatly increases the chance of a referral, even if that resource was not tailored for eating disorders. As part of the quantitative survey providers stated they would be very likely to refer identified eating disorder patients to existing social workers embedded in the practice, even though this resource is not set up to deliver eating disorder treatment. This speaks to the major demands on providers’ time that necessitate a convenient resource. Consequently, attempts to engage primary care providers in eating disorder management would be wise to rely on existing processes including convenient and onsite resources if at all possible.

Primary care providers particularly favored a design wherein a few colleagues in the practice are given extra training and identified as experts or “superusers” [22]. Specifically, this core team would provide assurance that the identifying provider will receive assistance throughout the treatment and the patient will not be the sole responsibility of the identifying provider. Having this core team could potentially improve screening and detection even without additional training for the practice. It is possible that, once a motivated few are in place as experts, confidence and knowledge of the entire practice could grow and fear might decrease. Importantly, this also serves to improve assessment and detection, because providers know they have a resource to which to refer if needed.

Findings must be interpreted within the context of several limitations. In particular, while the quantitative survey was sent to all primary care providers who worked with children at the 6 practices, concerns about maintaining confidentiality of respondents meant that differences between responders and nonresponders were not able to be evaluated. Though the quantitative and qualitative surveys were developed by content and methodological experts, we were unable to assess the validity of these measures. Further, providers self-selected for the qualitative interview, which may have influenced the generalizability of our results. Additionally, providers were all practicing in clinics serving largely rural or suburban populations, it is unclear how these results would generalize to providers in other settings, including those that serve within urban centers. Finally, results may be influenced by the particular treatment resources available to this sample of primary care provider participants. As noted, this medical center has numerous mental health resources, including embedded pediatric mental health services and social work and, as such, providers are perhaps more sensitized to the potential for integrating mental health intervention within the primary care setting.

## Conclusions

Despite these limitations, this study fills an important gap both in confirming primary care providers’ perceptions of the potential of primary care as a point of assessment and triage for young patients with eating disorders, and exploring barriers that need to be addressed in order to successfully integrate these services into the setting. Specialists in the field of eating disorders should attend to the findings that primary care providers see improving eating disorder detection and education as an area of need, and one that is important to their practice. Equally salient is the idea that all interventions are not created equal, and that attempts to leverage this setting to improve early detection and intervention for eating disorders must take into account the noted barriers and needs of the primary care setting. Further research is needed to develop programs of eating disorder education and intervention that meet the needs and fill the gaps identified by primary care providers and to examine the feasibility and efficacy of these programs for treating pediatric patients with eating disorders.

## Supplementary Information

**Additional file 1: Supplementary Material.** Quantitative Provider Survey.

**Additional file 2: Supplementary Material.** Provider Interview Guide.

## Data Availability

The datasets analyzed during the current study are available from the corresponding author on reasonable request.

## Abbreviations

CPAM: Community Pediatrics and Adolescent Medicine
EAT-26: Eating Attitudes Test
