# Supplementary material for: Engaging primary care providers in managing pediatric eating disorders: a mixed methods study
Source: J Eat Disord. 2021 Jan 14;9:11. doi: 10.1186/s40337-020-00363-8 (PMC7807397; doi:10.1186/s40337-020-00363-8)
Supplement: Supplementary file 1 — Additional file 1: Supplementary Material. Quantitative Provider Survey. [file 40337_2020_363_MOESM1_ESM.docx]

Supplementary Material. Brief Provider Opinion Survey

Please specify your current level of practice:

MD/DO Nurse Practitioner Physician’s Assistant

We are interested in your experience with child/adolescent eating issues.

For the purposes of this survey, when we say “disordered eating” we are referring to all levels of eating issues, including (but not limited to): picky eating, dieting behavior, restrictive eating, binge eating, purging, anorexia nervosa, bulimia nervosa, other specified feeding and eating disorders, and subclinical eating issues.

We are specifically asking about your experience at this time with disordered eating in both males and females, ages 18 years old and under.

1. You are seeing a 14-year-old girl who is new to you for a 30 minute appointment. She came in complaining of fatigue, with no other significant medical symptoms. Her PHQ-9 score is a 16, and she has noted that she “sometimes” has thoughts of death or dying. She has started running cross country and has eliminated gluten from her diet because she feels it makes her run slower. Her BMI has decreased from the 55^th^ percentile to the 35^th^ percentile. She reports feeling uninterested in socializing, has difficulties concentrating, and feels hopeless and disconnected when she thinks about her future.

How likely are you to refer this patient to:

Not Very

At All Likely Likely

IBH Social Work triage 1 2 3 4 5

Psychotherapy 1 2 3 4 5

Teen depression group 1 2 3 4 5

Dietician 1 2 3 4 5

Psychiatry 1 2 3 4 5

Eating disorder specialist 1 2 3 4 5

2. At this time, how confident are you in your ability to evaluate a patient for the presence of disordered eating in your daily practice?

1 2 3 4 5 6 7 8 9 10

Not Very At All Confident

Confident

3. At this time, how confident are you in your ability to manage disordered eating medically in your daily practice?

1 2 3 4 5 6 7 8 9 10

Not Very At All Confident

Confident

4. At this time, how confident are you in your ability to provide diagnostic feedback to families about the presence of an eating disorder/disordered eating in your daily practice?

1 2 3 4 5 6 7 8 9 10

Not Very At All Confident

Confident

1. At this time, how confident are you in your ability to advise and support a patient and their family in making decisions about his/her eating disorder treatment?

1 2 3 4 5 6 7 8 9 10

Not Very At All Confident

Confident

1. At this time, how many patients would you estimate you have treated with disordered eating (this includes patients for whom you’ve provided ongoing medical management for the disordered eating, referred for specialty eating treatment, provided feedback about eating to, or continued to see as part of your routine practice while they receive other treatment)?

1. At this time, how interested would you be in learning new tools or strategies for addressing disordered eating in your practice?

1 2 3 4 5 6 7 8 9 10

Not Very At All Interested

Interested

1. At this time, how interested would you be in the development of a new ICS for addressing eating and weight disorders?

1 2 3 4 5 6 7 8 9 10

Not Very At All Interested

Interested

If you are willing to be contacted for a brief (no more than 15 minutes) one-on-one interview as part of a needs assessment about eating disorder management in primary care, please put your name here, or contact Julie Hathaway via email at Hathaway.Julie@mayo.edu.
