# Supplementary material for: Engaging primary care providers in managing pediatric eating disorders: a mixed methods study
Source: J Eat Disord. 2021 Jan 14;9:11. doi: 10.1186/s40337-020-00363-8 (PMC7807397; doi:10.1186/s40337-020-00363-8)
Supplement: Supplementary file 2 — Additional file 2: Supplementary Material. Provider Interview Guide. [file 40337_2020_363_MOESM2_ESM.docx]

Supplementary Material. Provider Interview Guide

1. How much experience do you have with eating disorders?
   1. How many patients have you treated with eating disorders?
   2. What percentage of your panel would you guess has eating issues?
   3. How satisfied are you with the way eating disorders are addressed in your practice? Do you feel like you have the time and resources to address them as well as you would like?
   4. How interested would you be in new tools or strategies for addressing eating disorders in you practice?
2. What questions do you typically ask to screen for eating disorders?
   1. What tests do you order?
3. What are the signs that would make you suspect an eating disorder might be present?
4. What barriers are there to your screening for eating disorders regularly?
5. If you did not have access to a specialty care program for eating disorders (or there was a waitlist of several months for this type of program) what interventions, if any, would you do?
6. We are developing a primary care-based intervention for eating disorders. We have considered several models, including something similar to an ICS, or something like EMERALD. Do you have any preferences or ideas?
   1. How interested would you be in a program in which the primary care provider was responsible for weight restoration, with consultation from a psychologist and training on interventions?
      1. Ask about frequency of appointments, level of consultation needed (weekly huddle? On the fly?)
      2. What barriers do you see with this sort of program?
   2. What training would you be interested in or feel like you needed before you would feel comfortable taking the lead on a case like this?
   3. What services would you need to support this sort of a model (parent group? Booster trainings? Regular consultation group?)
